# A Study of the Origin and Nature of Pain1Read before the Pathological Section of the Buffalo Academy of Medicine, January 15, 1895.

**Published:** 1895-09

**Authors:** Herman G. Matzinger

**Affiliations:** Second Assistant Physician, Buffalo State Hospital


### A STUDY OF THE ORIGIN AND NATURE OF PAIN.[1]

By HERMAN G. MATZINGER, M. D.,
Second Assistant Physician, Buffalo State Hospital.

NO OTHER phenomena connected with the life history of the human body has been so great a factor in the historical development of medicine as pain. Indeed, it can readily be conceived that the first medical thought and effort was prompted by and directed to the relief of pain. And yet, though it is undoubtedly the most universal symptom of disease, there has been no entirely satisfactory explanation of its nature and mode of operation.

At present, two views are commonly held, one being the direct result of the insufficiency of the other, and both alike untenable because of lack of anatomical proof. The first was arrived at through the observation that pain is often associated with intense irritation of the end-organs of nerves of special sense, and holds that the sensation of pain differs only in degree from ordinary sensation; that the same nerve conveys both; that this is a common property of all the nerves of sensation, no matter what their special office may be.

There is, however, an unmistakable intrinsic difference between ordinary sensation and pain, which makes it impossible to accept that they are of the same nature. Both may occur together in the same place at the same time. If the greater irritation claims the operation of the nerve, how may it be that the lesser one is also conveyed and with equal certainty? Take, for instance, an inflamed condition of the skin associated with pain, and you will find that the tactile sense is not lost in the affected area. This observation led to the second view—namely, that there must be a special set of what might be termed pain nerves, running parallel and in the same trunk with the sensory nerves, having a special center

1. Read before the Pathological Section of the Buffalo Academy of Medicine, January 15, 1895.

of perception in the brain, and operating only under the influence of intense irritation.

But, anatomically, there is no basis for such a theory ; neither physiologically, since pain is a pathological phenomenon, and we have yet to find an organ, tissue or cell intended for anything but painless physiological functions. It is altogether unlikely, and contrary to natural laws, that there should be an elaborate mechanism of highly organised tissue, which is destined never to come into use in some individuals, or, at least, only in a very limited way.

Neither of these theories throws any light on the problem, so that we shall have to look elsewhere for a possible explanation.

Let us consider for a moment the conditions usually associated with pain and considered as pain-producing influences.

According to the first theory, an irritation of peripheral endings of sensory nerves, carried beyond a certain degree, produces pain. This might answer in parts where the nerve ends in tactile corpuscles, or some other special arrangement, but does not explain how the same irritation produces pain in tissues in which the nerve does not end in that way. The natural inference is, that only irritation of tissues containing special endings can be the seat of pain, which obviously does not hold.

Pressure is said to cause pain, and so it does, but the pain becomes more intense after the pressure is removed, and this is not due to injury to sensory nerve endings, since they functionate as well after the pressure is removed as before. Tumors often develop to great size and exert great pressure without producing pain.

It is well known that normal muscle action causes increased chemical changes in the muscle tissues, the products of which, under ordinary circumstances, are readily and promptly removed. But, if this becomes too rapid from over-exertion or prolonged exertion, the ordinary blood current is unequal to the task, transition products accumulate, and muscle tire is the result, which is quickly carried to the point of pain. This pain is not due to the pressure coincident to muscle contraction, because it is more intense when the muscle is at rest than when in action.

Pain has always been considered one of the cardinal symptoms of inflammation, and the tissue changes occurring during inflammatory processes are well known to be of the most profound nature. It is of special importance to note that during the early stages of inflammation, when the new products are being formed, embarrassing the local circulation, the pain is most intense, while after the exudate is collected, even in great quantities, it is not so intense, in spite of the great pressure. Many other illustrations could be mentioned, which seem to prove that pressure has little to do directly with pain, and that it is more likely to be the result of an abnormal, more or less intense, chemical tissue change in the part affected, which may be due to various causes, but is always associated with changes in the circulation in the part affected.

This holds good even for those portions of the body supplied with nerves of special sense. It was formerly thought that the pain produced by intense light was due to over-stimulation of the optic nerve-endings in the retina, but it is now universally accepted that the optic nerve is not directly affected by light, but through the medium of a photo-chemical change in the rods and cones, which, when carried too far, modifies or stops stimulation of the optic, recorded as vision, and produces pain. This is analogous to the phenomenon of muscle tire. Moreover, it has been observed that in conditions of total blindness, due to atrophy of the optic nerve, very intense light produced pain.

The pain associated with sunburn is due not to the degree of heat, but the action of the chemical rays of the spectrum. Electric light is especially irritating to the eye and skin, because it contains so large a proportion of violet rays. The degree of heat does not produce pain of itself until it either rises or falls to the point at which it produces protoplasmic changes.

Nor can the greater or lesser intensity of waves of sound be considered as producing earache or pain. The report of a cannon does not give us pain any more than the piping of a mouse. Loud and shrill sounds cause discomfort no more marked or disagreeable than the softest discord to the highly sensitive ear. If real pain be produced by violence of sound waves, it is more likely due to mechanical injury to the tympanum or oscicles, and continues long after the first cause has been removed.

The pain produced by the continuous electric current is easily explained by electrolitic cell changes, produced in nerve and tissues alike, while, if operating on the sensory nerves alone, sensation would only be produced by closing and breaking of the circuit, as is shown by its effect on motor nerves. The effect of the induced current is altogether different in this respect, and seems neither a sensation of touch, pressure nor pain. It may be a mixture of all, and, if continued long enough, may eventually produce real pain by working changes in cell nutrition.

Granting all this, it is not unreasonable to assume that pain originates in tissue change of a chemical nature, whose products are abnormal, at least in quantity, and that this change occurs principally in tissues other than nervous. But, to prove this, we must find some direct connection between them and the central nervous system.

We find that almost everywhere, but especially in connective tissue, there are numerous very fine simple nerve-endings that necessarily must be in connection with some large system of equally general distribution, and there is none other than the one that accompanies the blood-vessels—the vaso-motor system—that fills the requirements. Of these, the branches starting with the spinal nerves are the most suitable for study. The spinal nerves are mixed trunks, and contain besides motor and sensory fibers a third variety derived from the spinal ganglia of the sympathetic system, through their communicating branches. In their course to the periphery, these latter, unlike the other fibers, branch frequently in every direction, and follow the blood-vessels to their very smallest sub-divisions. They also form numerous plexuses containing ganglia of various sizes, some microscopic, consisting, apparently, of only one cell. These smallest ganglia lie close to, if not within, the adventitia, sending a fribrile to the media and one to the connective tissue cells in the immediate neighborhood, thus constituting a reflex arch in a way quite independent of the larger ganglia and the spinal cord (Kölliker), having what might be termed excito-motor functions and, it is not unreasonable to suppose, presiding over the life and nutrition of a given area of tissue; in other words, are minute trophic centers. They maintain a certain tone in the capillaries when chemical changes go on normally, like a mild continuous current, and, when the stimulus is increased, produce a dilatation of the vessels, *not* a contraction, as is noticed in muscle reflex.

This, in a way, explains the effect of cocaine, which is known to be a poison to all protoplasm, stopping metabolic changes, when injected, and so stopping the operation of this microscopic reflex arch, with the result that the tone producing and dilating influence of the vessel is lost and consequently a local ischemia is established, evidenced by the commonly observed blanching of the tissues and skin. Under such condition there is no record of chemical change made in the nervous center, no reflex ; consequently no sensation of pain, or even consciousness of existence.

A group of these ganglia, again, have a reflex center in higher ganglia, and these in the spinal cord, thus opening the way for inhibitory influences and a general effect—the consciousness of well-being, or discomfort, or pain.

It should be remarked here, that the vaso-motor constrictors exercise this inhibitory function. But they are independent of what might be called the true vaso-motors and issue from the anterior spinal root, run directly, without branching or entering the ganglia, to their respective fields of operation.

If these facts be true, there is every reason for believing that the entire phenomenon of pain is created and conveyed in the sympathetic vaso-motor system, as suggested by Oppenheimer.

It is, unfortunately, impossible to prove this theory by actual experiment, since section of the vaso-motor fibers alone in the spinal nerve trunk is out of the question, and section of the *rami communicantes* very difficult surgically. Even if this latter could be accomplished, there would be no certainty that all fibers are cut, because many may run to higher or lower spinal ganglia and thus communication with the cord not completely severed. But a number of physiological, pathological and anatomical observations may be mentioned, which seem to uphold the theory.

The various organs and tissue vary greatly in their capacity for pain and may be divided into three large groups : first, such as are always without pain, whether their condition be normal or abnormal ; second, such as are sensitive to pain in any condition of health or disease ; and, third, such as are the seat of pain only during profound pathological processes in them.

On first thought it would seem as though the number of centripetal or sensory nerve fibers in a tissue must determine, in some degree, its capacity for pain. But there are tissues apparently well supplied with such nerves, which nevertheless rarely exhibit pain. For instance, the tendons are well supplied with sensory nerves, with special end organs, that probably have to do with what is termed muscle sense. Still, they are rarely the seat of pain. So lung tissue is richly supplied with nerves from the pulmonary and cardiac plexuses, and must also have many centripetal fibers controlling respiration, and yet the lung proper is never the seat of pain. Pneumonia runs its full course without pain, unless the pleura is involved. Absolutely painless tissues are such as contain no nerves and, what is more important, no blood-vessels ; for example, cartilage, nails, hair, and the like.

A study of the arrangement of the blood-vessels in various tissues may throw more light on this difference in sensitiveness to pain. In all glandular organs there is a net-like distribution of vessels immediately beneath the membrana propria, with which the epithelial cells are in direct contact, while in all other tissues the vessels branch off in all directions independently of one another in the connective tissue, until they become capillaries and again veins. In the former case they have nothing to do with nutrition of tissue—are not so well supplied by vaso-motor nerves and thus are rarely the seat of pain, while in the latter they have no other function but nutrition—are exceedingly well supplied with vaso-motors, and report any marked disturbance of that function as pain.

The lung belongs to the former class. In its alveolar structure excretion and secretion go on as if it were without epithelial intervention, and its capillary circulation is exactly like that of other glands.

All such tissues are not sensitive to pain. Catarrhal inflammation of mucous membranes are painless ; so, too, parenchymatous nephritis, hepatitis, splenitis and adenitis.

Most sensitive to pain are the tissues that have a copious supply of blood-vessels in their connective tissue framework. They comprise the second class. This condition is most marked, therefore, in connective tissue enveloping special tissues, organs, bones, etc., like the periosteum, perimyseum, perineum, pleura and peritoneum. So we find that pain is invariably associated with inflammation of serous membranes, acute interstitial nephritis and perihepatitis, suppurating adenitis, periostitis and inflammatory rheumatism.

It would seem, then, that the question of whether a tissue can or cannot be painful depends on the vaso-motor nerve supply of its blood-vessels. Take, for example, bone, and its histology shows that the vessels entering the compact tissue lose their muscular coats, therefore have no vaso-motor nerves there. But the vessels of periosteum and marrow have them, are richly supplied with nerves and are sensitive, while compact bone is not. Periostitis and osteomyelitis are excruciatingly painful ; caries is not.

It has been demonstrated (Sappey) that the nerves entering the lung with the pulmonary artery are distributed only to the vessels of the bronchi, but no further. We know also that reflex nerve influences, which affect the blood pressure of the general arterial system, do not affect the pulmonary artery (Bradford). Only disturbances of respiration affect it ; therefore no pain in pneumonia. True, pain is experienced in inflammation of the bronchi, but they are supplied by the bronchial artery.

The pain of neuralgia is rather difficult to account for on this plan, because it is impossible to see the entire course of the nerve and thus to determine whether or not it is associated with changes in the circulation. Romberg and other observers have seen varicosities in the smaller vessels of the neurilemma, and still other authorities insist outright that neuralgia is accompanied with hyperemia and that the location of this change is indicated by the pressure points.

It remains to consider briefly the connection of the entire vaso-motor system with the cord. As has been mentioned before, the vaso-constrictors issue with the motor root and, like motor nerves proper, run directly to the periphery without communicating with the spinal ganglia or any other. Neither do they ever convey the sense of pain, no matter how they are stimulated or irritated. The sympathetic vaso-motors issue by the posterior root with the sensory fibers and enter the spinal ganglia. Their fibers are described as being smaller than the sensory, and it is not certain whether they are always non-medulated or not, as both varieties have been seen in the connecting branches between ganglia. In the cord they can be traced to the anterior lateral ascending tract, but some fibers also pass to the anterior horn, and still others to higher or lower ganglion cells.

I may mention here that the condition known as syringomyelia is, in a way, an excellent proof of this theory. There is also on record a case of glioma of the posterior horn of the cord in the cervical region, which caused degeneration of fibers in the anterior lateral ascending tract as high up as the olivary body, and which produced during life a swelling of the left forearm with redness and complete loss of sensibility to pain, but only modified the sense of touch.

We have a right, therefore, to conclude that there must be some connection between these cells and the periphery other than the sensory nerves, and which may be the true conductors of pain.

But it must not be lost sight of that this is only the smallest portion of their function, if, indeed, it can be called a function at all. Pain is a pathological phenomenon, and we have yet to find a single indication of any intention that any part of the economy is planned to do anything that is not strictly physiological and normal. It is, therefore, manifestly absurd to suppose that there is a special provision for the conversion of abnormal conditions into conscious experience. Pain was never intended to be a normal life experience, so pain nerves are not provided. Sensory nerves and nerves of special sense are intended solely for protection of the body from harm coming from without, and to put the mind into communication with the outer world, and this was not intended to be painful.

But everyone will admit that there is such a thing as a general sense of existence, a consciousness of living, of being well, which, modified, becomes a feeling of discomfort, whose superlative is pain and which may be independent of the operation of any or all the special senses. That this is due to cell metabolism, or the state of activity, nutrition and health, of the individual tissue cell. Life activity creates that general feeling of strength and pleasure known to those who work, but which becomes fatigue, discomfort and pain, too well known to those who overwork and abuse all or any special part of that "wonderfully and fearfully made" empire of the will—the human body.

